# *N*,*N*′-Bis(3-methyl­phen­yl)succinamide dihydrate

**DOI:** 10.1107/S1600536811020940

**Published:** 2011-06-04

**Authors:** B. S. Saraswathi, Sabine Foro, B. Thimme Gowda

**Affiliations:** a Department of Chemistry, Mangalore University, Mangalagangotri 574 199, Mangalore, India; b Institute of Materials Science, Darmstadt University of Technology, Petersenstrasse 23, D-64287 Darmstadt, Germany

## Abstract

The asymmetric unit of the title compound, C_18_H_20_N_2_O_2_·2H_2_O, contains half a mol­ecule with a center of symmetry at the mid-point of the central C—C bond. The N—H bonds in the amide fragments are *anti* to the *meta*-methyl groups in the adjacent benzene rings. The dihedral angle between the benzene ring and the NH—C(O)—CH_2_ segment in the two halves of the mol­ecule is 5.6 (4)°. In the crystal, the packing of mol­ecules through O—H⋯O and N—H⋯O hydrogen-bonding inter­actions leads to the formation of layers parallel to the *bc* plane. The methyl group is disordered with respect to the 3- and 5-positions of the benzene ring, with site-occupation factors of 0.910 (8) and 0.090 (8).

## Related literature

For the study of the effect of substituents on the structures of *N*-(ar­yl)-amides, see: Gowda *et al.* (2000 ▶); Saraswathi *et al.* (2011*a* ▶,*b* ▶). For the effect of substituents on the structures of *N*-(ar­yl)methane­sulfonamides, see: Gowda *et al.* (2007 ▶). For similar structures, see: Pierrot *et al.* (1984 ▶).
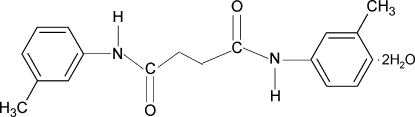


## Experimental

### 

#### Crystal data

C_18_H_20_N_2_O_2_·2H_2_O
*M* *_r_* = 332.39
Monoclinic, 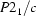

*a* = 13.401 (4) Å
*b* = 4.937 (2) Å
*c* = 14.446 (4) Å
β = 108.67 (3)°
*V* = 905.5 (5) Å^3^
*Z* = 2
Mo *K*α radiation
μ = 0.09 mm^−1^
*T* = 293 K
0.48 × 0.12 × 0.04 mm

#### Data collection

Oxford Diffraction Xcalibur diffractometer with a Sapphire CCD detector
Absorption correction: multi-scan (*CrysAlis RED*; Oxford Diffraction, 2009 ▶) *T*_min_ = 0.960, *T*_max_ = 0.997
2857 measured reflections
1679 independent reflections
797 reflections with *I* > 2σ(*I*)
*R*_int_ = 0.064

#### Refinement

*R*[*F* ^2^ > 2σ(*F* ^2^)] = 0.126
*wR*(*F* ^2^) = 0.159
*S* = 1.23
1679 reflections
123 parameters
10 restraints
H-atom parameters constrained
Δρ_max_ = 0.23 e Å^−3^
Δρ_min_ = −0.23 e Å^−3^

### 

Data collection: *CrysAlis CCD* (Oxford Diffraction, 2009 ▶); cell refinement: *CrysAlis RED* (Oxford Diffraction, 2009 ▶); data reduction: *CrysAlis RED*; program(s) used to solve structure: *SHELXS97* (Sheldrick, 2008 ▶); program(s) used to refine structure: *SHELXL97* (Sheldrick, 2008 ▶); molecular graphics: *PLATON* (Spek, 2009 ▶); software used to prepare material for publication: *SHELXL97*.

## Supplementary Material

Crystal structure: contains datablock(s) I, global. DOI: 10.1107/S1600536811020940/wm2494sup1.cif

Structure factors: contains datablock(s) I. DOI: 10.1107/S1600536811020940/wm2494Isup2.hkl

Supplementary material file. DOI: 10.1107/S1600536811020940/wm2494Isup3.cml

Additional supplementary materials:  crystallographic information; 3D view; checkCIF report

## Figures and Tables

**Table 1:** Hydrogen-bond geometry (Å, °)

*D*—H⋯*A*	*D*—H	H⋯*A*	*D*⋯*A*	*D*—H⋯*A*
N1—H1N⋯O2^i^	0.86	2.10	2.946 (6)	169
O2—H21⋯O2^ii^	0.82	2.08	2.836 (4)	153
O2—H22⋯O1	0.84	1.87	2.713 (5)	178

Symmetry codes: (i) 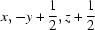; (ii) 
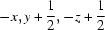
.

**Fig. 1.**
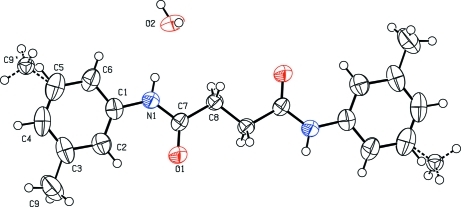
Molecular structure of the title compound, showing the atom labelling scheme and displacement ellipsoids are drawn at the 50% probability level. The disordered methyl group at the 3 and 5 positions of the phenyl ring is shown with both orientations.

**Fig. 2.**
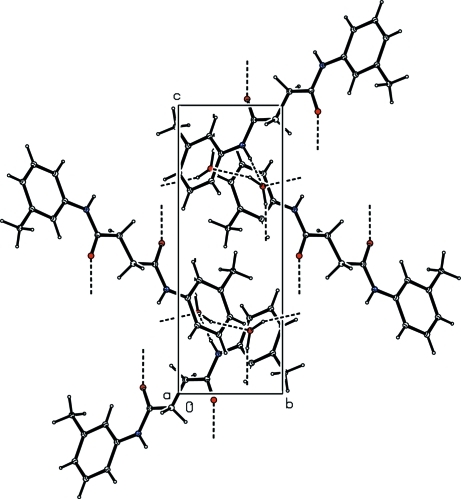
Molecular packing of the title compound with hydrogen bonding shown as dashed lines.

## supplementary crystallographic information

### Comment

The amide and sulfonamide moieties are important constituents of many biologically significant compounds. As a part of studying the substituent effects on the structures of this class of compounds (Gowda *et al.*, 2000, 2007; Saraswathi *et al.*, 2011**a*, b*), in the present work, the structure of *N*,*N*-bis(3-methylphenyl)-succinamide dihydrate, (I), has been determined (Fig.1). The asymmetric unit of (I) contains half a molecule with a center of symmetry at the mid-point of the central C—C bond, similar to that observed in bis(2-chlorophenylaminocarbonylmethyl)disulfide, (II), (Pierrot *et al.*, 1984), *N*,*N*-bis(2-methylphenyl)- succinamide, (III), (Saraswathi *et al.*, 2011*a*) *N*,*N*- bis(3-chlorophenyl)-succinamide (IV) (Saraswathi *et al.*, 2011*b*).

In the C—NH—C(O)—C segment, the amide O atom is *anti* to the H atoms attached to the adjacent C atom. The N—H bonds in the amide fragments are also *anti* to the *meta*-methyl groups in the adjacent benzene rings, similar to that observed with respect to the *ortho*-methyl groups in (III) and the *meta*-chloro groups in (IV).

The dihedral angle between the benzene ring and the NH—C(O)—CH_2_ segment in the two halves of the molecule is 5.6 (4)°, compared to the values of 62.1 (2)° in (III) and 32.8 (1)° in (IV). The striking difference may be due to the fact that the title compound is the dihydrate, *i.e.* is composed of the amide and lattice water molecules, which, unlike in other compounds, influence the molecular conformation through hydrogen bonding interactions.

The torsion angles of N1–C7–C8–C8a and O1–C7–C8–C8a in (I) are 175.9 (6)° and -5.3 (9)°, compared to the values of 150.9 (3)° and -30.5 (4)° in (III) and -175.4 (2)° and 5.9 (4)° in (IV). The differences in the torsion angles may be due to the steric hindrances caused by the different substituents.

Similarly, the torsion angles of C2—C1—N1—C7 and C6—C1—N1—C7 are 5.4 (9)° and -173.6 (6)°, compared to the values of -64.0 (4)° and 117.6 (3)° in (III) and -35.0 (3)° and 147.5 (2)° in (IV).

The crystal packing of (I), through N1—H1N···O2, O2—H21···O2 and O2—H22···O1 hydrogen bonding (Table 1), leads to the formation of layers parallel to the *bc* plane and is shown in Fig. 2.

### Experimental

Succinic anhydride (0.01 mol) in toluene (25 ml) was treated dropwise with *m*-toluidine (0.01 mol) also in toluene (20 ml) with constant stirring. The resulting mixture was stirred for one hour and set aside for an additional hour at room temperature for completion of the reaction. The mixture was then treated with dilute hydrochloric acid to remove unreacted *m*-toluidine. The resultant *N*-(3-methylphenyl)succinamic acid was filtered under suction and washed thoroughly with water to remove the unreacted succinic anhydride and succinic acid. The compound was recrystallized to a constant melting point from ethanol. The purity of the compound was checked by elemental analysis and characterized by its infrared and NMR spectra.

The *N*-(3-methylphenyl)succinamic acid obtained was then treated with phosphorous oxychloride and excess of *m*-toluidine at room temperature with constant stirring. The resultant mixture was stirred for 4 h, kept aside for additional 6 h for completion of the reaction and poured slowly into crushed ice with constant stirring. It was kept aside for a day. The resultant solid, *N*,*N*-bis(3-methylphenyl)-succinamide dihydrate, was filtered under sucction, washed thoroughly with water, dilute sodium hydroxide solution and finally with water. It was recrystallized to a constant melting point from a mixture of acetone and chloroform. The purity of the compound was checked by elemental analysis, and characterized by its infrared and NMR spectra.

Needle-like colorless single crystals used in the X-ray diffraction studies were grown in a mixture of acetone and chloroform at room temperature.

### Refinement

The H atom of the NH group was located in a difference map and later restrained to the distance N—H = 0.86 (2) Å. The stucture was modelled with stoichiometric chemical composition applying an approximation of full occupancy of H5 and no corresponding partly occupied hydrogen atom at C3. The C9'H3 group in an alternative orientation was idealized and refined using a AFIX 3 (positional optimization of the entire group only by translation, no rotations) in SHELXL. The U^ij^ components of C9' were assumed to be identical with that of C5 (EADP C5 C9') and were restrained to approximate isotropic behaviour. Atom C9 was refined using a split model. The corresponding site-occupation factors were refined so that their sum was unity [0.910 (8) and 0.090 (8)]. A DELU restraint was used for all U^ij^. The water molecule was refined as a rigid group with respect to *x*,y,*z* and its orientation (AFIX 6). The other H atoms were positioned with idealized geometry using a riding model with the aromatic C—H = 0.93 Å, the methyl C—H = 0.96 Å and the methylene C—H = 0.97 Å. *U*_iso_(H) values of the methyl group and the water molecule were set at 1.5 *U*_eq_ of the parent atom. The other H atoms were refined with isotropic displacement parameters (set to 1.2 times of the *U*_eq_ of the parent atom).

The crystals available for X-ray studies were of rather poor quality and weak scatterers at high theta value resulting in relatively high *R* values.

### Crystal data

**Table:**

C_18_H_20_N_2_O_2_·2H_2_O	*F*(000) = 356
*M**_r_* = 332.39	*D*_x_ = 1.219 Mg m^−^^3^
Monoclinic, *P*2_1_/*c*	Mo *K*α radiation, λ = 0.71073 Å
Hall symbol: -P 2ybc	Cell parameters from 433 reflections
*a* = 13.401 (4) Å	θ = 2.9–28.2°
*b* = 4.937 (2) Å	µ = 0.09 mm^−^^1^
*c* = 14.446 (4) Å	*T* = 293 K
β = 108.67 (3)°	Needle, colourless
*V* = 905.5 (5) Å^3^	0.48 × 0.12 × 0.04 mm
*Z* = 2	

### Data collection

**Table:**

Oxford Diffraction Xcalibur diffractometer with a Sapphire CCD detector	1679 independent reflections
Radiation source: fine-focus sealed tube	797 reflections with *I* > 2σ(*I*)
graphite	*R*_int_ = 0.064
Rotation method data acquisition using ω scans	θ_max_ = 25.7°, θ_min_ = 2.9°
Absorption correction: multi-scan (*CrysAlis RED*; Oxford Diffraction, 2009)	*h* = −10→16
*T*_min_ = 0.960, *T*_max_ = 0.997	*k* = −6→4
2857 measured reflections	*l* = −17→17

### Refinement

**Table:**

Refinement on *F*^2^	Primary atom site location: structure-invariant direct methods
Least-squares matrix: full	Secondary atom site location: difference Fourier map
*R*[*F*^2^ > 2σ(*F*^2^)] = 0.126	Hydrogen site location: inferred from neighbouring sites
*wR*(*F*^2^) = 0.159	H-atom parameters constrained
*S* = 1.23	*w* = 1/[σ^2^(*F*_o_^2^) + (0.*P*)^2^ + 1.5559*P*] where *P* = (*F*_o_^2^ + 2*F*_c_^2^)/3
1679 reflections	(Δ/σ)_max_ = 0.008
123 parameters	Δρ_max_ = 0.23 e Å^−^^3^
10 restraints	Δρ_min_ = −0.23 e Å^−^^3^

### Special details

**Table:**

Geometry. All e.s.d.'s (except the e.s.d. in the dihedral angle between two l.s. planes) are estimated using the full covariance matrix. The cell e.s.d.'s are taken into account individually in the estimation of e.s.d.'s in distances, angles and torsion angles; correlations between e.s.d.'s in cell parameters are only used when they are defined by crystal symmetry. An approximate (isotropic) treatment of cell e.s.d.'s is used for estimating e.s.d.'s involving l.s. planes.
Refinement. Refinement of *F*^2^ against ALL reflections. The weighted *R*-factor *wR* and goodness of fit *S* are based on *F*^2^, conventional *R*-factors *R* are based on *F*, with *F* set to zero for negative *F*^2^. The threshold expression of *F*^2^ > σ(*F*^2^) is used only for calculating *R*-factors(gt) *etc*. and is not relevant to the choice of reflections for refinement. *R*-factors based on *F*^2^ are statistically about twice as large as those based on *F*, and *R*- factors based on ALL data will be even larger.

### Fractional atomic coordinates and isotropic or equivalent isotropic displacement parameters (Å^2^)

**Table:**

	*x*	*y*	*z*	*U*_iso_*/*U*_eq_	Occ. (<1)
O1	0.1286 (3)	0.1598 (8)	0.4781 (2)	0.0590 (12)	
N1	0.1521 (3)	0.1149 (9)	0.6385 (3)	0.0471 (13)	
H1N	0.1313	0.1791	0.6846	0.056*	
C1	0.2292 (4)	−0.0896 (12)	0.6675 (4)	0.0467 (15)	
C2	0.2784 (4)	−0.2019 (13)	0.6062 (4)	0.0582 (17)	
H2	0.2614	−0.1412	0.5421	0.070*	
C3	0.3535 (5)	−0.4063 (13)	0.6396 (6)	0.0668 (19)	
C4	0.3776 (5)	−0.4944 (14)	0.7340 (6)	0.076 (2)	
H4	0.4281	−0.6288	0.7572	0.091*	
C5	0.3280 (5)	−0.3861 (15)	0.7938 (6)	0.081 (2)	
H5	0.3439	−0.4510	0.8574	0.098*	
C6	0.2547 (5)	−0.1828 (13)	0.7627 (4)	0.0625 (18)	
H6	0.2226	−0.1086	0.8051	0.075*	
C7	0.1061 (4)	0.2251 (11)	0.5504 (4)	0.0408 (14)	
C8	0.0227 (4)	0.4323 (11)	0.5484 (3)	0.0407 (14)	
H8A	−0.0337	0.3441	0.5655	0.049*	
H8B	0.0530	0.5691	0.5975	0.049*	
C9	0.4042 (6)	−0.5247 (16)	0.5729 (5)	0.098 (3)	0.910 (8)
H9A	0.4538	−0.3979	0.5624	0.147*	0.910 (8)
H9B	0.3516	−0.5661	0.5116	0.147*	0.910 (8)
H9C	0.4403	−0.6878	0.6010	0.147*	0.910 (8)
C9'	0.367 (4)	−0.529 (11)	0.878 (4)	0.041 (18)	0.090 (8)
H9'A	0.4200	−0.6566	0.8750	0.061*	0.090 (8)
H9'B	0.3956	−0.4078	0.9317	0.061*	0.090 (8)
H9'C	0.3080	−0.6241	0.8861	0.061*	0.090 (8)
O2	0.0524 (3)	0.1884 (9)	0.2803 (2)	0.0583 (12)	
H21	0.0046	0.3006	0.2619	0.087*	
H22	0.0756	0.1747	0.3415	0.087*	

### Atomic displacement parameters (Å^2^)

**Table:**

	*U*^11^	*U*^22^	*U*^33^	*U*^12^	*U*^13^	*U*^23^
O1	0.071 (3)	0.067 (3)	0.038 (2)	0.022 (2)	0.016 (2)	−0.002 (2)
N1	0.054 (3)	0.047 (3)	0.040 (3)	0.010 (3)	0.015 (2)	−0.002 (2)
C1	0.038 (3)	0.038 (4)	0.057 (4)	−0.006 (3)	0.006 (3)	−0.005 (3)
C2	0.052 (4)	0.055 (4)	0.067 (4)	0.000 (4)	0.018 (3)	0.002 (4)
C3	0.043 (4)	0.053 (5)	0.101 (6)	0.005 (4)	0.018 (4)	0.003 (4)
C4	0.057 (5)	0.056 (5)	0.098 (6)	0.010 (4)	0.001 (4)	0.020 (5)
C5	0.080 (6)	0.070 (6)	0.080 (5)	0.009 (5)	0.006 (4)	0.023 (5)
C6	0.067 (4)	0.058 (5)	0.054 (4)	0.007 (4)	0.007 (3)	0.009 (4)
C7	0.046 (4)	0.035 (4)	0.037 (3)	−0.004 (3)	0.007 (3)	0.000 (3)
C8	0.051 (3)	0.035 (4)	0.036 (3)	0.006 (3)	0.014 (3)	−0.001 (3)
C9	0.086 (6)	0.099 (7)	0.121 (7)	0.037 (5)	0.051 (5)	0.005 (6)
C9'	0.041 (18)	0.040 (19)	0.040 (18)	0.000 (5)	0.013 (7)	0.001 (5)
O2	0.079 (3)	0.062 (3)	0.037 (2)	0.012 (2)	0.023 (2)	0.008 (2)

### Geometric parameters (Å, °)

**Table:**

O1—C7	1.219 (5)	C6—H6	0.9300
N1—C7	1.340 (6)	C7—C8	1.509 (6)
N1—C1	1.409 (6)	C8—C8^i^	1.492 (8)
N1—H1N	0.8600	C8—H8A	0.9700
C1—C2	1.378 (7)	C8—H8B	0.9700
C1—C6	1.385 (7)	C9—H9A	0.9600
C2—C3	1.398 (8)	C9—H9B	0.9600
C2—H2	0.9300	C9—H9C	0.9600
C3—C4	1.368 (8)	C9'—H9'A	0.9600
C3—C9	1.466 (8)	C9'—H9'B	0.9600
C4—C5	1.358 (8)	C9'—H9'C	0.9600
C4—H4	0.9300	O2—H21	0.8235
C5—C6	1.375 (8)	O2—H22	0.8398
C5—H5	0.9300		
			
C7—N1—C1	130.1 (5)	C1—C6—H6	120.3
C7—N1—H1N	115.0	O1—C7—N1	122.8 (5)
C1—N1—H1N	115.0	O1—C7—C8	123.2 (5)
C2—C1—C6	119.1 (6)	N1—C7—C8	114.0 (5)
C2—C1—N1	123.6 (5)	C8^i^—C8—C7	113.5 (5)
C6—C1—N1	117.2 (5)	C8^i^—C8—H8A	108.9
C1—C2—C3	120.6 (6)	C7—C8—H8A	108.9
C1—C2—H2	119.7	C8^i^—C8—H8B	108.9
C3—C2—H2	119.7	C7—C8—H8B	108.9
C4—C3—C2	119.2 (6)	H8A—C8—H8B	107.7
C4—C3—C9	121.1 (7)	C3—C9—H9A	109.5
C2—C3—C9	119.7 (7)	C3—C9—H9B	109.5
C5—C4—C3	120.1 (7)	H9A—C9—H9B	109.5
C5—C4—H4	119.9	C3—C9—H9C	109.5
C3—C4—H4	119.9	H9A—C9—H9C	109.5
C4—C5—C6	121.5 (7)	H9B—C9—H9C	109.5
C4—C5—H5	119.2	H9'A—C9'—H9'B	109.5
C6—C5—H5	119.2	H9'A—C9'—H9'C	109.5
C5—C6—C1	119.4 (6)	H9'B—C9'—H9'C	109.5
C5—C6—H6	120.3	H21—O2—H22	112.4
			
C7—N1—C1—C2	5.4 (9)	C3—C4—C5—C6	−1.5 (11)
C7—N1—C1—C6	−173.6 (5)	C4—C5—C6—C1	1.3 (10)
C6—C1—C2—C3	−0.3 (8)	C2—C1—C6—C5	−0.4 (9)
N1—C1—C2—C3	−179.4 (5)	N1—C1—C6—C5	178.7 (5)
C1—C2—C3—C4	0.1 (9)	C1—N1—C7—O1	−1.5 (9)
C1—C2—C3—C9	179.4 (6)	C1—N1—C7—C8	177.3 (5)
C2—C3—C4—C5	0.8 (10)	O1—C7—C8—C8^i^	−5.3 (9)
C9—C3—C4—C5	−178.5 (7)	N1—C7—C8—C8^i^	175.9 (6)

Symmetry codes: (i) −*x*, −*y*+1, −*z*+1.

### Hydrogen-bond geometry (Å, °)

**Table:**

*D*—H···*A*	*D*—H	H···*A*	*D*···*A*	*D*—H···*A*
N1—H1N···O2^ii^	0.86	2.10	2.946 (6)	169.
O2—H21···O2^iii^	0.82	2.08	2.836 (4)	153.
O2—H22···O1	0.84	1.87	2.713 (5)	178.

Symmetry codes: (ii) *x*, −*y*+1/2, *z*+1/2; (iii) −*x*, *y*+1/2, −*z*+1/2.
